# Supplementary material for: Characterization of the IAA-Producing and -Degrading Pseudomonas Strains Regulating Growth of the Common Duckweed (Lemna minor L.)
Source: Int J Mol Sci. 2023 Dec 6;24(24):17207. doi: 10.3390/ijms242417207 (PMC10742903; doi:10.3390/ijms242417207)
Supplement: Supplementary file 1 [file ijms-24-17207-s001.zip › ijms-2661703-supplementary.pdf]

Materials and methods

S1. Bacterial production of IAA in the presence of 0.1% tryptophan

Bacterial strains with ability to produce IAA and to grow on minimal M9 medium with IAA as the sole carbon source were treated with Salkowski reagent as previously described (Section 2.2.). Absorbance was measured spectrophotometrically at 536 nm on Agilent 8453 UV/Visible Spectrophotometer (Agilent, USA). Measurements were done in triplicates. Results were presented graphically in Microsoft Word Excel (2010).

S2. Bacterial Growth in Luria – Bertani (LB) medium

In order to obtain information on growth kinetics of selected bacteria in rich LB nutrient medium, the bacterial growth curve was constructed for each strain following the standard growth curve protocol. A single colony of each strain was inoculated in 5 ml sterile Luria-Bertani (LB) and incubated overnight at +30°C. The 1 ml of each overnight culture was transferred into 49 ml sterile LB and the samples for zero hour were taken immediately, after which the cultures were incubated in thermoshaker at +30°C. Sampling was performed every two hours (up to 12th hour of incubation), as well as determination of OD_{600} and spread plating on LB medium in appropriate dilutions (from 10^{-4} to 10^{-8}, respectively). The spread plating, as well as all spectrophotometric measurements, were performed in triplicates. Generation times and growth rates were calculated in Microsoft Word Excel (2010).

Results

Addition of tryptophan (0.1% w/v) induced more IAA production in *P. oryzihabitans* D1-104/3 and *P. putida* A3-104/5 compared to growth in LB without exogenously added L-Trp (Figure S1), which suggested that IAA production in these strains is stimulated (upregulated) in the presence of L-Trp (Figure S1). Production of IAA was increased more than 10 times for *P. oryzihabitans* D1-104/3 and almost 3 times for *P. putida* A3-104/5 when 0.1% L-Trp was added, respectively. For *P. gessardii* C31-106/3 and *P. yamanorum* C44-104/1, no such increase was measured, i.e., while *P. gessardii* C31-106/3 produced (13.9 ± 0.1) mg l^{-1} without L-Trp, with L-Trp it produced (17.4 ± 0.2) mg l^{-1} of IAA. Similarly, *P. yamanorum* C44-104/1 produced (20.8 ± 0.2) mg l^{-1} without L-Trp, and (22.4 ± 0.2) mg l^{-1} with L-Trp.

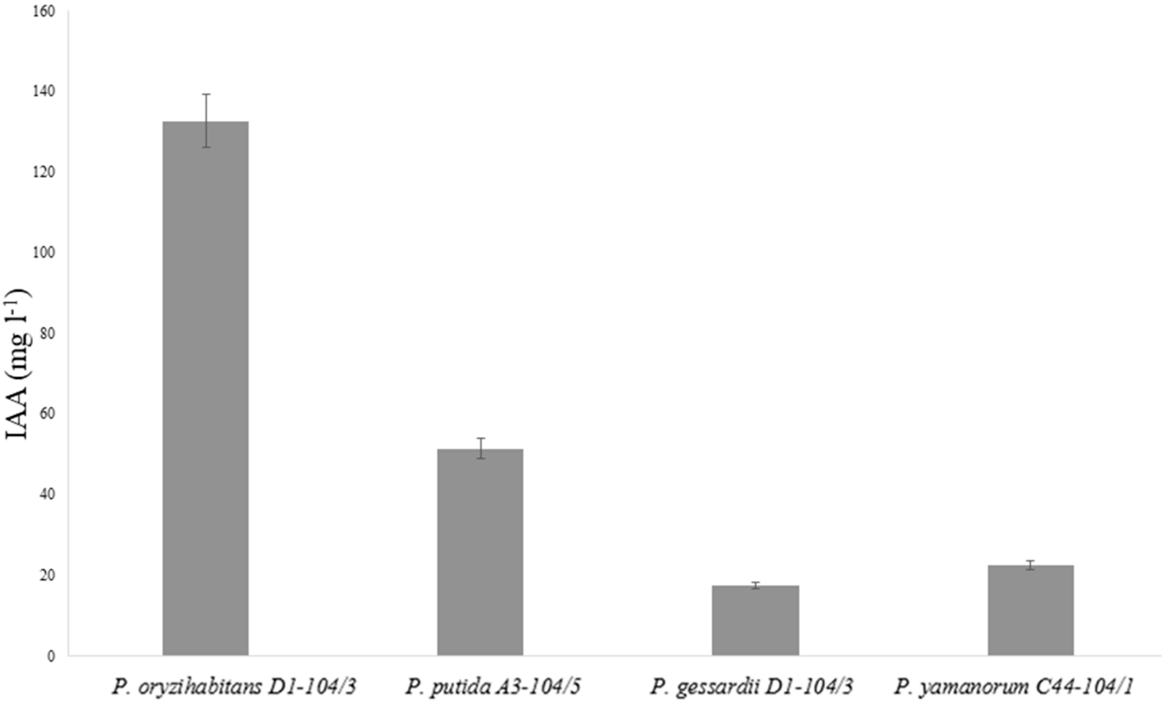

Figure S1. Bacterial production of IAA with exogenous L-Trp.

Bacterial Growth Curve

CFU ml^{-1} values for each time point were calculated from the mean value of dilution plates. For the easier interpretation, the results are expressed in logarithmic values. The lag phase extends out to around 2h of growth; transitions to exponential phase from 2h to 12h, and then enters the stationary phase. (Table S1, Figure S2).

Table S1. CFU ml^{-1} per hour, expressed in logarithmic values.

Strain	0h	2h	4h	6h	8h	10h	12h
P. oryzihabitans D1-104/3	6.66 ± 0.01	8.15 ± 0.03	9.45 ± 0.12	9.59 ± 0.01	10.41 ± 0.03	11.34 ± 0.05	12.01 ± 0.01
P. putida A3-104/5	7.90 ± 0.01	8.22 ± 0.01	9.83 ± 0.01	9.36 ± 0.06	10.50 ± 0.04	10.87 ± 0.03	12.72 ± 0.03
P. gessardii C31-106/3	7.92 ± 0.03	8.15 ± 0.05	9.13 ± 0.01	9.80 ± 0.01	10.71 ± 0.04	12.09 ± 0.03	12.07 ± 0.04
P. yamanorum C44-104/1	6.00 ± 0.01	7.01 ± 0.11	8.43 ± 0.02	9.49 ± 0.02	8.86 ± 0.07	9.20 ± 0.17	9.59 ± 0.11

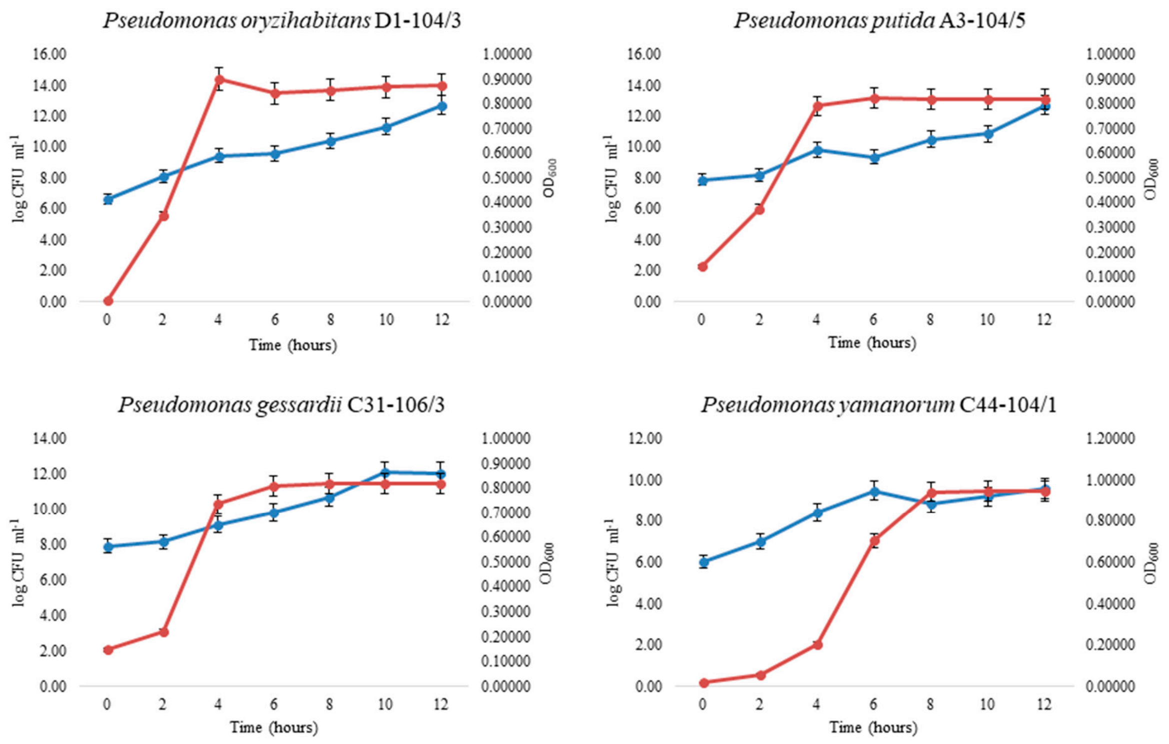

Figure S2. Bacterial growth curve for CFU ml^{-1} versus OD$_{600}$. The optical density was used to calculate approximate bacterial cell number (log CFU ml $^{-1}$ per hour is presented as blue curve, while OD was presented as red).

P. gessardii C31-106/3 had the longest generation time (and lowest growth rate) in liquid LB medium compared to other bacterial strains.

Table S2. Generation time and growth rate of bacterial strains grown in liquid LB medium.

Strain	Generation time (h)	Growth rate (h^{-1})
P. oryzihabitans D1-104/3	0.51 ± 0.01	1.37 ± 0.02
P. putida A3-104/5	1.62 ± 0.08	0.43 ± 0.02
P. gessardii C31-106/3	5.37 ± 0.05	0.13 ± 0.01
P. yamanorum C44-104/1	1.12 ± 0.05	0.62 ± 0.05

Co-cultivation of bacteria and duckweeds

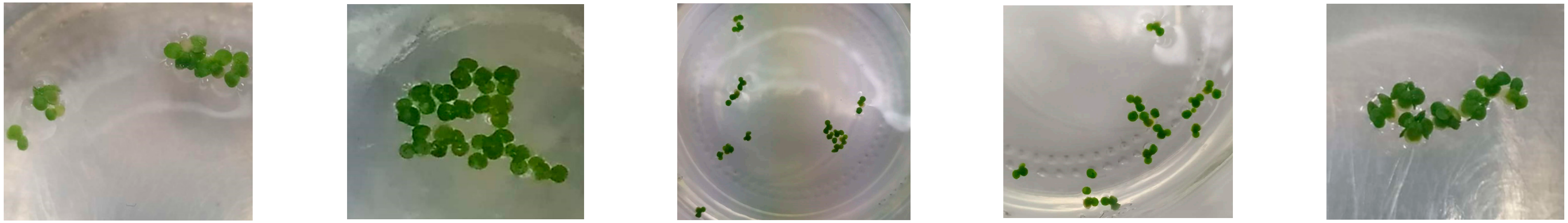

(a) **(b)** **(c)** **(d)** **(e)**

Figure S3. Duckweed growth in presence of the selected strains after 7 days of incubation, without addition of IAA. (a) *P. oryzihabitans* D1-104/3; (b) *P. putida* A3-104/5; (c) *P. gessardii* C31-106/3; (d) *P. yamanorum* C44-104/1; (e) negative control
